# A Feasibility and Efficacy Randomized Controlled Trial of an Online Preventative Program for Childhood Obesity: Protocol for the EMPOWER Intervention

**DOI:** 10.2196/resprot.2141

**Published:** 2012-06-21

**Authors:** Adam Knowlden, Manoj Sharma

**Affiliations:** 1 Health Promotion & Education ProgramUniversity of CincinnatiCincinnati, OHUnited States

**Keywords:** Pediatrics, child, social cognitive theory, family, overweight, obesity, program, intervention

## Abstract

Background: The home and family environment is a highly influential psychosocial antecedent of pediatric obesity. Implementation of conventional family- and home-based childhood obesity interventions is challenging for parents, often requiring them to attend multiple educational sessions. Attrition rates for traditional interventions are frequently high due to competing demands for parents’ time. Under such constraints, an Internet-based intervention has the potential to modify determinants of childhood obesity while making judicious use of parents’ time. Theory-based interventions offer many advantages over atheoretical interventions, including reduced intervention dosage, increased likelihood of behavioral change, and efficient resource allocation. Social cognitive theory (SCT) is a robust theoretical framework for addressing childhood obesity. SCT is a behavior change model rooted in reciprocal determinism, a causal paradigm that states that human functioning is the product of a dynamic interplay of behavioral, personal, and environmental factors. Objectives: To evaluate the efficacy of the Enabling Mothers to Prevent Childhood Obesity Through Web-Based Education and Reciprocal Determinism (EMPOWER) program, an Internet-based, theory-driven intervention for preventing childhood overweight and obesity. The project goal is supported by two specific aims: (1) modification of four obesogenic protective factors related to childhood obesity (minutes engaged in physical activity, servings of fruits and vegetables consumed, servings of sugar-sweetened and sugar-free beverages consumed, and minutes engaged in screen time), and (2) reification of five maternal-mediated constructs of SCT (environment, expectations, emotional coping, self-control, and self-efficacy). Methods: We will recruit mothers with children ages 4 to 6 years from childcare centers and randomly assign them to either the theory-based (experimental) or knowledge-based (control) arm of the trial. Data for the intervention will be collected at three intervals: baseline (week 0), posttest (week 4), and follow-up (8 weeks). At each phase of data collection, we will collect from both groups (1) measures of the four obesogenic protective factors, and (2) summated SCT construct scores. Constructs will be measured by a psychometrically valid and reliable SCT-based instrument. Behaviors will be evaluated by a behavior log. We will use a repeated-measures one-between-, one-within-participants design to evaluate intervention results. Constructs will be modified through Web-based learning modules, online interactive worksheets, and mother–child home-based activities. Process evaluation will assess program fidelity.

## Introduction

Since 1980 the prevalence of childhood obesity has tripled among school-age children and adolescents in the United States [1]. Etiologically, pediatric obesity is strongly influenced by the environment [2]. Researchers have attempted to modify antecedents of childhood obesity in a variety of environmental contexts, including communities, schools, and after-school programs [3]. From an ecological perspective, the home and family environment is perhaps the most influential psychosocial milieu of pediatric obesity. Parents play an integral role in shaping the eating and exercise behaviors of developing children [4]. Furthermore, children consume an estimated two-thirds of their dietary intake within the home environment [5]. As a result, there has been an increased call for interventions that target young children within the context of the home and family environment [6,7].

However, unlike alternative intervention formats that focus on children as the salient agents of change, researchers implementing family- and home-based interventions have discovered that targeting parents exclusively produces more significant outcomes [8-16]. Within the family and home context, there has been a greater focus on targeting mothers as the primary change agents. It has been well established that overweight and obesity disproportionally affect low-income and minority children. Subsequently, there has been a natural proclivity to integrate family- and home-based obesity programming with maternal-based, government-funded nutrition programs such as the Special Supplemental Nutrition Program for Women, Infants, and Children [17]. Additionally, there is evidence that maternal behaviors including gestational weight gain and smoking contribute to higher risk of pediatric obesity [18]. Breastfeeding has also been cited as a protective factor against childhood obesity [19].

While there is little dispute that parents are powerful change agents in the lives of young children, focusing solely on parents presents several challenges. These barriers must be addressed if family- and home-based interventions are to be accepted as an effective preventative treatment for childhood overweight and obesity. The Enabling Mothers to Prevent Childhood Obesity Through Web-Based Education and Reciprocal Determinism (EMPOWER) program will address four salient barriers confronting the advancement of family- and home-based childhood overweight and obesity interventions: (1) efficacy of theory-based family- and home-based interventions, (2) deficit in theoretical construct measurement, (3) low program participation and high attrition rates, and (4) absence of intervention implementation process evaluation.

### Efficacy of Theory-Based Interventions

We systematically reviewed randomized control trial family- and home-based interventions targeting children ages 2 to 7 years and found that only 3 of the 9 interventions applied a theoretical framework [20]. It is generally recognized that theory-based interventions are more efficacious than atheoretical interventions [21]. The economic advantages of theory-based interventions are primarily attributed to design and measurement efficiencies. Measurement tools for theory-based interventions are developed through psychometric modeling. Validated theoretical models can provide detailed insight into the dynamics that underlie behavior change. By reifying the specific theoretical constructs that predict a given behavior, interventionists can design programs that are more likely to result in behavior modification. To evaluate the efficacy of theory-based family- and home-based interventions, our project will compare the social cognitive theory (SCT)-based EMPOWER intervention (experimental) with an equivalent atheoretical, knowledge-based intervention (control).

### Deficit in Theoretical Construct Measurement

Although theory-based interventions can produce significant outcome measures, it is critical that interventionists operationalize the theories they employ. Concurrently, researchers must measure changes in the reified constructs from before to after the intervention using psychometrically valid instruments. In doing so, they can evaluate theoretical constructs and advance more effective intervention designs. Without adequate measurement, it becomes difficult to determine which mediating variables were attributed to the positive health outcomes of the intervention. Among the family- and home-based theory-based interventions, we were unable to confirm any that operationalized or measured the constructs of the theoretical frameworks the researchers applied. The current investigation will overcome this deficit by operationalizing and measuring five constructs of SCT.

### Low Program Participation and High Attrition Rates

An additional barrier to family- and home-based interventions is the time commitment required of parents to participate in face-to-face educational sessions. Researchers employing family- and home-based interventions have commented that programs emphasizing these modalities have resulted in lower recruitment and retention rates [22]. To maximize participation and retention, our study will employ Web-based learning as the primary catalyst of behavior modification. Online learning offers flexibility and convenience to parents. It also promotes self-paced learning and skill mastery. An Internet-based, theory-driven family- and home-based intervention offers the potential to improve outcome measures, minimize overhead costs, foster program replication, and enable wide-scale intervention dissemination.

### Absence of Process Evaluation of Intervention Implementation

Process evaluation is applied in interventions to evaluate program fidelity, dose, reach, stakeholder satisfaction, and exposure of the intervention modalities. Only two of the family- and home-based interventions we reviewed incorporated process evaluation [10,13]. Among these cases, we were unable to verify any that incorporated intervention implementation process evaluation. Implementation process evaluation is a specific type of process evaluation that examines fidelity of program delivery. Assessment of implementation allows the researchers to ensure the program was delivered to the participants in the prescribed fashion. Failure to evaluate program fidelity can make it difficult to confirm whether nonsignificant program outcomes were due to ineffective intervention components or inadequate transference of intervention deliverables. Our protocol will employ several layers of intervention implementation process evaluation to properly assess outcome measures.

### Specific Aims

#### Specific Aim 1

The American Medical Association, in collaboration with 15 health care organizations, developed a series of recommendations for the prevention and treatment of child and adolescent obesity [23]. The key lifestyle behaviors identified by the committee will serve as the basis for the behavioral outcome measures of this study. The four obesogenic protective factors measured in the intervention will be the child’s (1) engagement in 60 minutes of moderate to vigorously intense physical activity each day, (2) consumption of 3 or more cups of fruits and vegetables each day, (3) replacement of sugar-sweetened beverages with sugar-free beverages, and (4) limitation of screen time (television/computer time) to no more than 2 hours per day.

Therefore, the first specific aim of this study is to compare the effects of the EMPOWER intervention (experimental) with an equivalent knowledge-based intervention (control) on the four identified obesogenic protective factors from baseline (week 0) to postintervention (week 4) and 1-month follow-up (week 8) in children ages 4 to 6 years as measured through a valid and reliable behavior log.

#### Specific Aim 2

SCT is a robust theoretical framework for eliciting behavior change [24]. SCT is based on the premise of reciprocal determinism, a causal model that posits that human functioning is the result of environmental, personal, and behavioral factors. SCT is rooted in human potential and emphasizes modeling, symbolizing, forethought, self-regulatory, and self-efficacy capabilities. The key constructs of SCT will serve as the theoretical outcome measures for the study. The five SCT constructs that will be operationalized for the EMPOWER program will be (1) environment, which comprises the external stimuli to which an individual is exposed, (2) expectations, which are the anticipations that an individual has about a specific outcome and the values the individual places on those outcomes, (3) emotional coping, which is an individual’s ability to control emotional and psychological states associated with acquisition of a new behavior, (4) self-control, which results from explicit and specific goal setting for accomplishing a behavior, and (5) self-efficacy, which results from confidence in personal capability to perform a behavior. 

The second specific aim of this study is to compare the effects of the EMPOWER intervention (experimental) with a knowledge-based intervention (control) on the five SCT maternal-mediated constructs from baseline (week 0) to postintervention (week 4) and 1-month follow-up (week 8) in mothers of children ages 4 to 6 years as measured by a SCT-based instrument.

## Methods

### Instrumentation

In support of specific aims 1 and 2, we are collecting data to develop an instrument capable of measuring the five listed maternal-mediated SCT constructs for predicting the four obesogenic protective factors outlined in this study. Instrumentation will encompass three stages of data collection and analysis. Stage 1 is complete and included evaluation of face and content validity of the instrument by a panel of six experts over two rounds, in addition to readability assessment by the Flesch Reading Ease Test and Flesch-Kincaid Grade Level Test [25]. The instrument was word processed in Microsoft Word Professional 2010 (Microsoft Corporation, Redmond, WA, USA) and analyzed by the software’s readability statistics function. Based on the findings of the software, the instrument had a Flesch Reading Ease Test score of 72%. Scores ranging from 70% to 79% are categorized as “fairly easy” in terms of reading ease [25]. The software populated a Flesch-Kincaid Grade Level test score of 5.7, indicating the reading level of the instrument was between a fifth- and sixth-grade school reading level according to US educational standards [26].

Stage 2 will assess the test–retest reliability of the instrument by having the same group of participants (n = 30) complete the instrument two separate times with 4 weeks between administrations. We have set acceptable test–retest coefficient values a priori at 0.70. Stage 3 will evaluate construct and predictive validity of the instrument through structural equation modeling. Each of the four behavioral determinants of childhood obesity (endogenous variables) will be modeled separately according to the five SCT constructs (exogenous variables). Applying a participant to parameter ratio of 5:1, we will require a sample size of 165 to build each model. The final specified models will serve as the theoretical framework for designing and measuring the EMPOWER intervention.

### Interventions

**Figure 1:**
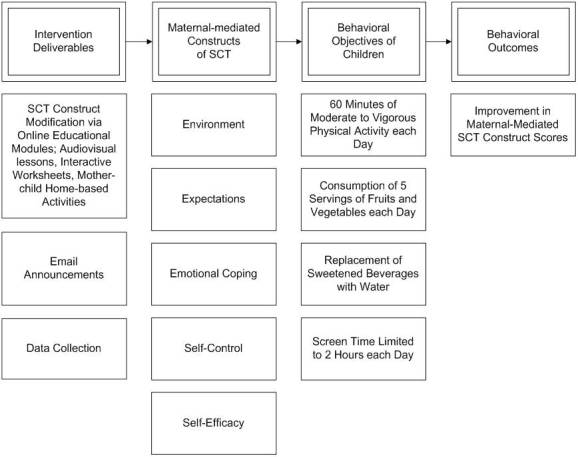
Logic model for the Enabling Mothers to Prevent Childhood Obesity Through Web-Based Education and Reciprocal Determinism (EMPOWER) intervention. SCT = social cognitive theory.

The most innovative component of the intervention is the novel medium of delivery (see Figure 1). Despite the highly promising potential of Web-based programs, this modality has not been adequately tested and reported in the childhood obesity intervention literature. The proposed brief intervention will span 2 months. The four obesogenic protective factors will be targeted through weekly online modules for a total of four modules. The Internet-based program will be hosted on the University of Cincinnati’s Blackboard Version 9 (Blackboard Inc, Washington, DC, USA) platform. Each week participants will be sent email messages to promote program involvement and participation.

#### Theory-Based Intervention (Experimental)

Each of the four modules for the experimental group will incorporate affective, cognitive, and experiential pedagogical approaches to promote maternal-mediated behavior change in children based on the five selected SCT constructs. Module curricula will include 15-minute audiovisual sessions developed and produced by the research team. Interactive online worksheets and newsletters will complement each module. The interactive worksheets will be designed to reinforce the concepts conveyed in the audiovisual sessions. Self-efficacy for the four obesogenic protective factors will be built through home-based activities completed by participating mothers and their children.

#### Knowledge-Based Intervention (Control)

The complementary and equivalent knowledge-based program will also target the four obesogenic protective factors; however, the control program will not focus on modification of SCT constructs. Instead, the program will center on delivery of general health knowledge regarding the four obesogenic protective factors as opposed to theory-based behavior modification. Newsletters and audiovisual sessions will complement each module, but we will not include interactive components designed to increase self-efficacy in the mothers.

### Intervention Design and Sampling

#### Design

**Figure 2:**
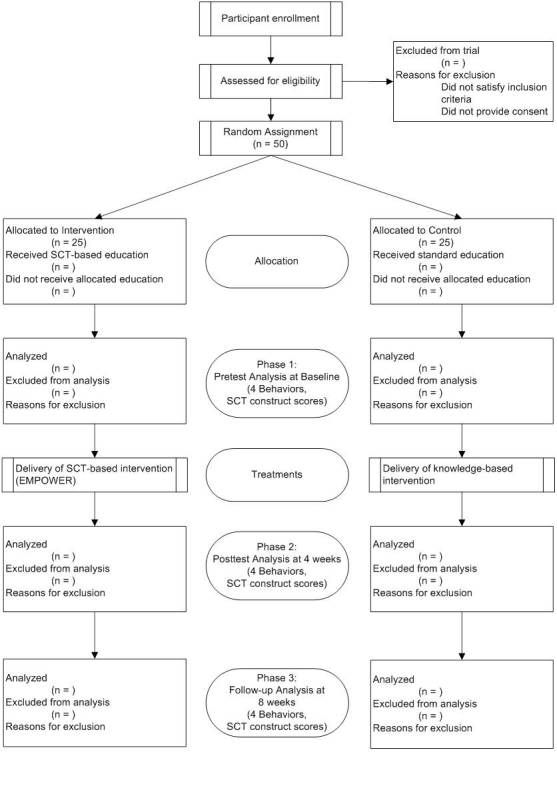
Flow of participants through the randomized control trial. EMPOWER = Enabling Mothers to Prevent Childhood Obesity Through Web-Based Education and Reciprocal Determinism, SCT = social cognitive theory.

The proposed 8-week program will use a group randomized experimental design to test the efficacy of the protocol. Statistically, we will use a repeated-measures one-between-, one-within-participants design. The protocol will be delivered to two cohorts randomly assigned to receive either the theory-based (experimental) or knowledge-based (control) protocol (see Figure 2). The primary independent variable in the proposed study is the intervention. This is a fixed, categorical variable with two levels: EMPOWER intervention and knowledge-based intervention. The second independent variable in the study is time (within-participants effect). This is also a fixed, categorical variable with three levels: (1) baseline, (2) postintervention (at 4 weeks), and (3) 1-month follow-up (8 weeks after baseline). The outcome measures for comparison between the experimental and control groups are the four obesogenic protective factors and the five SCT constructs.

#### Sample Size for Efficacy Trial

Based on previous studies, significance criteria for this investigation were set at an alpha of .05, a power at 80%, and an effect size at 0.20 [12,14,27]. We established the number of groups at 2 and the number of measurements at 3. Correlation between repeated measures was set at .05 and nonsphericity correction at 1. Inputting these criteria into G*Power resulted in a sample size of 42 [28]. To account for potential attrition, we inflated the sample size by 20%, resulting in a total sample size of 50 with 25 participants allocated to each group.

### Participant Enrollment

We selected mothers as the primary agents of change in this intervention [29-33]. Children ages 4 to 6 have been targeted for this study because this age range has been demonstrated to be a strong predictor of future health [30,34]. Eligibility to participate in the intervention will be limited to the following inclusion criteria: (1) mothers with (2) high-speed Internet access, (3) working email, and (4) at least one child in the age range of 4 to 6 years. Exclusion criteria will be (1) non-English-speaking mothers, (2) pregnant mothers, and mothers with (3) a child outside the age range of 4 to 6 years, (4) a child inside the age range of 4 to 6 years with a physical disability that would interfere with participating in daily moderate to vigorously intense physical activity, (5) a child with a medical condition associated with weight gain, (6) a child prescribed medication associated with weight gain, or (7) a child enrolled in an additional weight management program. We will seek and obtain university institutional review board approval before implementing the intervention. Eligible mothers will be required to provide consent prior to enrollment. Children will be required to provide oral assent prior to enrollment.

### Participant Recruitment

The intervention will target the general population in Cincinnati, Ohio, USA. We will recruit 50 families from 10 to 15 local childcare centers (preschools, daycares, and kindergartens) to participate in the investigation [15]. Childcare center recruitment will entail calling local childcare center managers and requesting their support for the initiative. Presentations will be made to the childcare center managers describing the benefits of the program. During the presentations, we will emphasize the childcare centers’ role as a community stakeholder to garner support. Participant recruitment activities will include delivering flyers and 15-minute presentations to potential recipients at the childcare centers.

### Random Assignment

Eligible, consenting participants will be randomly assigned to either the theory-based (experimental) or knowledge-based (control) arm of the trial. The participant assignment protocol will be based on block randomization (block size will remain confidential to maintain allocation blindness). Allocation of block assignment will be determined using random number generator computer software. Participants will not know to which cohort they have been assigned to improve external validity. However, as we will recruit participants within the same childcare facilities, it is possible there will be spillover effects between the intervention and control groups. For example, mothers in the intervention group may share what they are learning with mothers in the control group. To buffer this effect, we will ask participants not discuss intervention-based activities with others in the facility. Maintenance of confidentially will be evaluated through process evaluation at the beginning and end of the intervention.

### Process Evaluation

Process evaluation will occur concurrently with each module to assess program fidelity and dose. Process evaluation is akin to quality control, in which standardized methods are employed to ensure programming is delivered systematically to all participants. In the absence of process evaluation, researchers increase the risk of committing a type III error. From a quality control perspective, a type III error occurs when weak or null results occur due to inconsistent or inadequate intervention delivery. To reduce the risk of committing a type III error, the proposed intervention will incorporate several layers of implementation process evaluation:

- Log-in codes and tracking data will be used to assess whether the website and subsequent module materials were accessed.
- Date and duration of activity will be logged to assess whether audiovisuals were viewed and adequate time was spent to complete each activity.
- Online, interactive worksheets and module quizzes will have forced-response validation to gauge transference of information.
- Reminder emails will be sent to assess promotion.
- At the completion of the intervention, respondents will be requested to complete an open-ended questionnaire regarding acceptability and perceived usefulness of the program. Additionally, data regarding maintenance of confidentiality will be collected.
- Participants who drop out of the program will be contacted and asked why they discontinued the intervention. To help control for attrition the sample size has been inflated by 20% above what was required by the study power analysis.

### Data Analysis

We will collect data for the intervention over three phases: phase 1 at baseline (week 0), phase 2 at posttest (week 4), and phase 3 at follow-up (week 8). At each phase of data collection, we will collect from both groups (1) measures of the four obesogenic protective factors, and (2) summated scores for the SCT constructs. SAS version 9.3 (SAS Institute, Cary, NC, USA) will be used to conduct statistical analyses of the intervention. Each of the outcome measures will be tested using PROC MIXED to analyze the data collected in the repeated-measures statistical design. The model will account for gender (male, female) as a potential covariate.

The null hypothesis for the time effect is that the means of the outcome measures at each of the three levels will be equal. If we find statistically significant differences, we will plot least square means and apply the Scheffé post hoc test. Normality of the distribution of outcome measures, homogeneity of variances, and the Mauchly test of sphericity will be used to test the assumptions of repeated-measures analysis of variance. Variables that are not normally distributed or that display variance heterogeneity will be transformed to obtain distributions that are closer to normality and variance homogeneity. The Huynh and Feldt adjustment to any *F*-distribution degrees of freedom will be applied if the covariances between the repeated measures being analyzed do not follow a spherical distribution.

## Discussion

The proposed project will advance the fields of public health, health promotion, and health education through the following means.

### Optimization of Multicomponent Programs

While many interventionists employ theories of human behavior in designing their programs, few measure and evaluate the changes in the theoretical constructs they apply. The current proposal will employ construct operationalization and intervention delivery process evaluation to identify which constructs of the SCT have the largest impact on obesogenic protective factors in the target population. Optimization of SCT constructs will assist health practitioners in shaping public health policy, facilitating resource allocation for future interventions, and fostering educational advancements.

### Intervention Delivery

Implementation of traditional family- and home-based interventions in the target population is challenging for parents, often requiring them to attend numerous educational sessions. Even with incentives, attrition rates for traditional interventions are often high due to competing demands for parents’ time. Given the current economic climate, time constraints are likely to intensify for parents, making traditional intervention delivery more problematic. Under such constraints, an Internet-based intervention has the potential to modify antecedents of childhood obesity while making judicious use of parents’ time. An efficacious online, theory-driven, family- and home-based intervention will offer health practitioners a novel vehicle for addressing childhood overweight and obesity in the target population.

### Limitations of the Proposed Protocol

The proposed protocol is not without limitations. Analysis of the study’s specific aims is limited by the self-reporting accuracy, integrity, and honesty of the participants. Participants will be requested to provide information on the four obesogenic protective factors and constructs of SCT. Inaccuracy in memory or misinterpretation of instrument items may skew the final analysis. Even with accurate self-reporting, the proposed timeline may not be sufficient to detect statistically significant differences in behaviors from before to after the intervention. Additionally, the four obesogenic protective behaviors will be based on 24-hour recall, which may not be representative of a typical day.

Participation in this study will be voluntary and will require both the parent’s and child’s assent. Childhood obesity is a sensitive topic, and some parents may not wish to have their child participate, believing that involvement in such an intervention speaks ill of their parenting skills. Children may not wish to participate for various reasons such as embarrassment or a lack of interest. In the current proposal, we target mothers as the primary agent of change. This is in tandem with the majority of family- and home-based interventions; however, researchers are increasingly calling for more involvement of fathers and grandparents [35]. Limiting the intervention only to mothers increases efficiency for a pilot study but invariably is not inclusive of the entire familial environment.

## Abbreviations

EMPOWER: Enabling Mothers to Prevent Childhood Obesity Through Web-Based Education and Reciprocal Determinism
SCT: social cognitive theory
